# BRG1 Promotes chromatin remodeling around DNA damage sites

**DOI:** 10.1080/19768354.2018.1525429

**Published:** 2018-10-01

**Authors:** Wenjing Qi, Hongyu Chen, Chengwen Lu, Qingpan Bu, Xiaoguang Wang, Liping Han

**Affiliations:** a Department of Bioscience, Changchun Normal University, Changchun, People’s Republic of China; b The Key Laboratory of Molecular Epigenetics of MOE, Institute of Genetics and Cytology, School of Life Sciences, Northeast Normal University, Changchun, People’s Republic of China

**Keywords:** BRG1, chromatin remodeling, DNA damage repair

## Abstract

Chromatin remodeling complexes play important roles in various DNA metabolism processes, including DNA damage repair. BRG1 is the core subunit of the SWI/SNF complex, which plays critical roles in cell cycle regulation, cell development, cell differentiation, and tumorigenesis. In the present study, we report that BRG1 depletion increased the percentage of apoptotic cells in etoposide-treated cells. Moreover, western blotting and immunofluorescence data showed that BRG1 depletion decreased H2AX phosphorylation and caused defective phosphorylated histone H2AX (*γ*H2AX) clearance. Furthermore, we found that in both SW13 and U2OS cells, BRG1 expression could increase the sensitivity of genomic DNA to micrococcal nuclease (MNase) and facilitate chromatin relaxation around DNA damage sites. Thus, the results provide evidence that BRG1 plays an important role in early DNA damage repair by remodeling the chromatin structure near DNA damage sites.

## 1. Introduction

In eukaryocytes, DNA damage is caused by endogenous and exogenous factors, such as chemotherapeutic agents and radiation. DNA integrity is critical for cell viability. The DNA damage repair process requires the concerted action of multiple proteins that sense the damage, transduce the signal, and finally execute the repair. The signals are believed to be sensed by the histone variant H2AX, which is phosphorylated (also known as *γ*H2AX) in response to DNA damage (Fillingham et al. 2006; Kuo and Yang 2008). It is clear that nucleosomes provide a barrier to DNA end processing, and this barrier must be removed for end resection to occur (Mimitou and Symington 2011). Increasing evidence has demonstrated that chromatin structure modulators participate in DNA damage repair (Mimitou and Symington 2009; Price and D’Andrea 2013; Gursoy-Yuzugullu et al. 2016).

BRG1 is a key component of the mammalian (m) SWI/SNF chromatin remodeling complex, which repositions nucleosomes using the energy released by ATP hydrolysis (Ho and Crabtree 2010). BRG1 might suppress malignancy through maintaining genome integrity because the loss of BRG1 generates spontaneous anaphase bridges (Dykhuizen et al. 2013). A recent study shows that BRG1 is recruited to DNA double-strand break (DSB) sites by interacting with *γ*H2AX-containing nucleosomes. This interaction involves the binding of the bromodomain of BRG1 to acetylated H3, which in turn induces more H3 acetylation through recruiting the acetyltransferase GCN5 (Lee et al. 2010). Additionally, BRG1 is needed to form non-coding RNA and 53BP1 foci in response to doxorubicin-induced DNA damage (Patne et al. 2017). Our previous study shows that BRG1 promotes DSB repair by facilitating the replacement of RPA with RAD51 at DSB sites (Qi et al. 2015). However, the specific function of BRG1 in chromatin remodeling near DNA damage sites remains obscure.

In the current study, we found that BRG1 depletion increased the percentage of cell apoptosis in etoposide-treated cells. Moreover, western blotting and immunofluorescence analyses showed that BRG1 depletion decreased H2AX phosphorylation and impaired H2AX dephosphorylation. Furthermore, we found that heterogenous BRG1 expression could facilitate chromatin relaxation around DNA damage sites and increase micrococcal nuclease (MNase) sensitivity in SW13 cells. Additionally, in BRG1 knockdown U2OS cells, the chromatin around DNA damage sites showed less relaxation and sensitivity to MNase. Therefore, the results provide evidence that BRG1 plays an important role in early damage repair by remodeling the chromatin structure near DNA damage sites.

## 2. Materials and methods

### 2.1. Antibodies and reagents

An anti-BRG1 antibody (ab70558) was purchased from Abcam (San Francisco, CA). Anti-*γ*H2AX (05-636) and anti-H2A (07-146) antibodies were obtained from Merck Millipore (CA). An anti-actin antibody (A5441) and MNase were purchased from Sigma Aldrich (St Louis, MO). Puromycin, etoposide (ETO), and 4-hydroxy tamoxifen (4OHT) were obtained from Sigma Aldrich (St Louis, MO).

### 2.2. Cell culture

U2OS (American Type Culture Collection) and Asi-ER-U2OS cells were grown in DMEM containing 10% FBS. SW13 (human adrenal adenocarcinoma cells) cells were cultivated in IMDM containing 10% FBS. All cells were cultivated at 37°C and 5% CO_2_ in a humidified incubator.

### 2.3. Plasmids and plasmid transfection

The pBABE HA-ER-AsiSI plasmid was generously provided by Gaelle Legube (Laboratoire de Biologie Cellulaire et Moleculaire du Controle de la Proliferation). The pBJ5-vector, pBJ5-wt BRG1 and ATPase mutant (K798R) BRG1 were generously provided by Anthony N. Imbalzano (University of Massachusetts Medical School). The pCBA-HA-Sce and vector plasmids were kindly provided by Xingzhi Xu (Capital Normal University).

Plasmid transfection was performed using Lipofectamine™ 2000 (Invitrogen, USA) according to the manufacturer’s instructions. Briefly, the transfections were performed in 60-mm dishes containing 70% confluent cells with 4 µg DNA per dish. Asi-U2OS cells stably transfected with the pBABE HA-AsiSI-ER plasmid were selected using 1 mg/ml puromycin. Isolated individual Asi-U2OS clones were further validated by western blotting.

### 2.4. RNA interference

siRNA duplex oligoribonucleotides were synthesized by GenePharma (China). siRNA duplex transfection was performed using Lipofectamine™ 2000 (Invitrogen, USA) according to the manufacturer’s instructions. The sense sequences for control and BRG1 siRNAs are shown in Table 1.

**Table 1.** Sequences for the primers and oligonucleotides used in this study.

Primer/siRNA name	Sequence
chr22:19180307_dist	FW: CCCATCTCAACCTCCACACT REV: CTTGTCCAGATTCGCTGTGA
chr22:19180307_prox	FW: CCTTCTTTCCCAGTGGTTCA REV: GTGGTCTGACCCAGAGTGGT
chr1_6:89231183	FW: GATTGGCTATGGGTGTGGAC REV: CATCCTTGCAAACCAGTCCT
chr6_12:90404906	FW: TGCCGGTCTCCTAGAAGTTG REV: GCGCTTGATTTCCCTGAGT
siBRG1 #3	Sense oligo: 5-UGG AGA AGC AGC AGA AGA UU-3
siBRG1 #5	Sense oligo: 5-CGA CGU ACG AGU ACA UCA UTT-3
siControl	Sense oligo: 5-UUC UCC GAA CGU GUC ACG UTT-3

### 2.5. Western blotting analysis

Cells in 100-mm dishes were washed with 1 × PBS, harvested and lysed in RIPA lysis buffer (50 mM Tris-HCl, pH 7.4, 150 mM NaCl, 1% Triton X-100, 1% sodium deoxycholate, 1% SDS, 1 mM EDTA, 1 mM Na_3_VO_4_, 2 mM NaF, 1 mM β-glycerophosphate, and 2.5 mM sodium pyrophosphate) containing 1 mM PMSF and protease inhibitors. These lysates were subjected to western blotting according to a previously reported protocol (Qi et al. 2015).

### 2.6. Immunofluorescence analysis

Cells were seeded on a coverslip 24 h prior to the experiment. The cells were treated with ETO for 20 min and then allowed to repair for the indicated times. The cells were rinsed with cold PBS, fixed in 10% formaldehyde for 15 min at room temperature, permeabilized with 0.5% Triton X-100 for 5 min, and blocked with 10% FBS for 45 min. The cells were incubated with primary antibodies for 1 h, washed with PBS, and counter-stained for 1 h with fluorochrome-conjugated secondary antibodies. The coverslips were stained with DAPI (0.4 mg/ml in PBS) and observed under a fluorescence microscope. The signal intensity of *γ*H2AX was scored qualitatively using ImageJ software as follows. The background signals were measured in three non-*γ*H2AX regions, and the average background values were subtracted from the nuclei values in the *γ*H2AX channels. The data collected included the average H2AX signal intensity per nucleus, which provides quantitative estimates of the DNA damage repair level.

### 2.7. Nucleosome stability assay

Cells were harvested, washed twice with ice-cold PBS and centrifuged at 1,000 rpm. The cell pellets were completely resuspended in 500 µl buffer A (20 mM Hepes, pH 7.9, 0.5 mM DTT, 1 mM PMSF, 1.5 mM MgCl_2_, and 0.1% Triton) containing 0.15 M NaCl to remove the cell membrane and cytosolic contents. The nuclear fraction was then collected by centrifugation and incubated in buffer A with 0.2–1.0 M NaCl for 40 min at 4°C with constant agitation. The samples were then centrifuged at 100,000 g (Ultracentrifuge; Beckman Coulter) for 20 min, and the supernatant containing the released histones was retained for western blotting.

### 2.8. Micrococcal nuclease (MNase) susceptibility assay

MNase assays were performed as described previously (Tsukuda et al. 2005). Briefly, cultured cells were harvested, centrifuged, and resuspended in ice-cold NP-40 lysis buffer (10 mM Tris-HCl, 10 mM NaCl, 3 mM MgCl_2_, 0.5% NP-40, 0.15 mM spermine, and 0.5 mM spermidine) for 5 min. The resultant nuclei were pelleted and resuspended in MNase digestion buffer (10 mM Tris-HCl, pH 7.4, 15 mM NaCl, 60 mM KCl, 0.15 mM spermine and 0.5 mM spermidine) containing 1 mM CaCl_2_. An undigested fraction of chromatin was stored at −20°C. The rest of the chromatin was subjected to digestion with 2 U, 5 U and 10 U of the MNase enzyme for 5 min at room temperature. The reaction was stopped by the addition of MNase stop buffer (15 mM EDTA and 2.3 mM EGTA). The digested samples were then treated with 60 μl 10% (v/v) SDS, 10 mg/ml proteinase K and 10 μl of 10 mg/ml RNase for 15 min at 37°C. DNA was extracted using Tris-saturated phenol and chloroform. Finally, the DNA was ethanol precipitated and resuspended in TE. The MNase-treated nucleosomal DNA was resolved on 1% agarose gels, visualized with ethidium bromide, and photographed with an Eagle Eye apparatus (Speed Light/BT Sciencetech-LT1000).

### 2.9. Chromatin immunoprecipitation assay

Cells were grown to 90% confluency in 100-mm cell culture dishes, and chromatin immunoprecipitation was performed using a previously published protocol (Qi et al. 2015). The immunoprecipitated and input DNA were analyzed in duplicate by real-time q-PCR using Platinum SYBR Green qPCR SuperMix (Invitrogen) according to the manufacturer’s instructions. Each experiment was carried out in triplicate.

### 2.10. Statistical analysis

All experiments were carried out in triplicate, and statistical analyses were performed in GraphPad 6 using an unpaired t-test when comparing two samples and one-way ANOVA when comparing more than two samples. Significance was determined with *P* < 0.05 (∗) indicating a significant difference and *P* < 0.01 (∗∗) indicating a highly significant difference.

### 3. Results

### 3.1. BRG1 depletion leads to significant ETO-induced cell apoptosis

**Figure 1.**
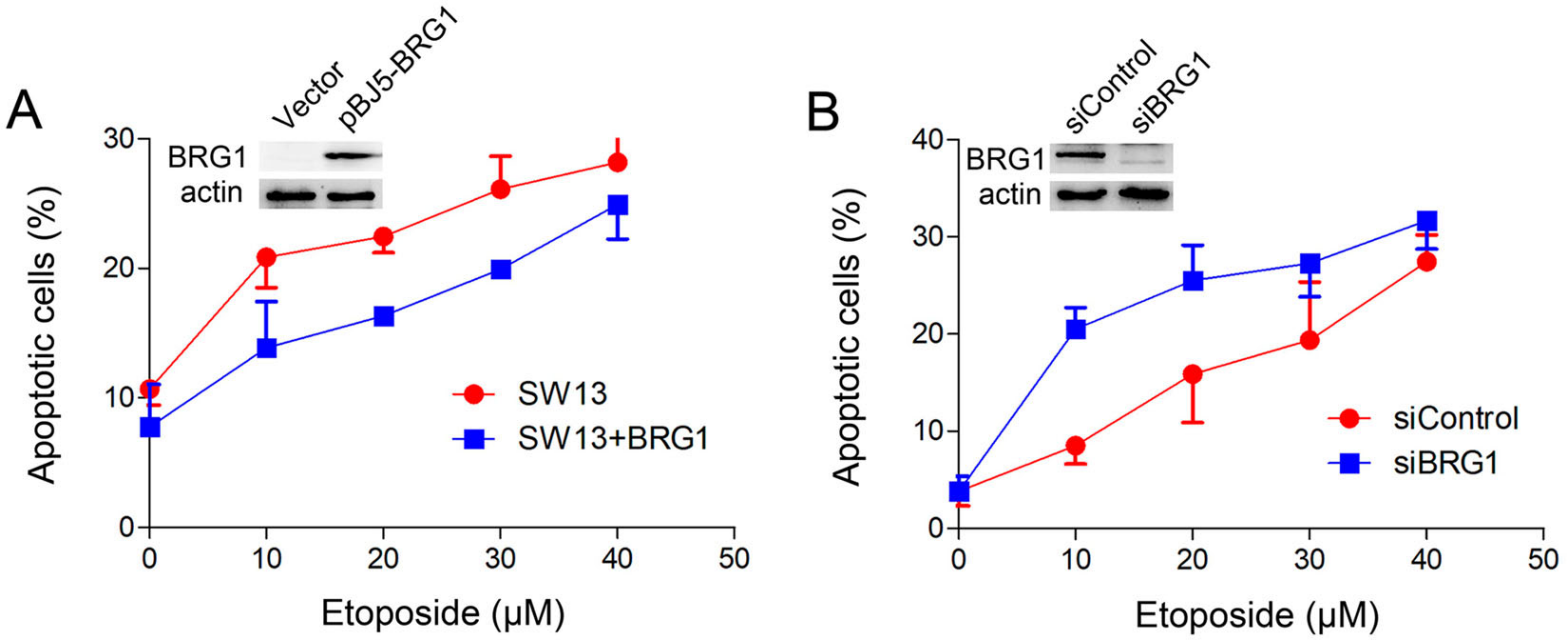
BRG1 depletion increases the percentage of apoptotic cells induced by ETO treatment. (A) SW13 cells were transfected with the pBJ5 empty vector or pBJ5-BRG1. Forty-eight hours later, the cells were treated with the indicated concentrations of ETO for 20 min, and apoptotic cells were detected by flow cytometry 24 h later. The percentage of apoptotic cells was analyzed by GraphPad 6. (B) U2OS cells were transfected with control siRNA or BRG1 siRNA for 48 h. Then, the cells were treated with the indicated concentrations of ETO for 20 min, and apoptotic cells were detected by flow cytometry 24 h later. The percentage of apoptotic cells was analyzed by GraphPad 6. The expression of BRG1 was analyzed by western blotting.

Previous studies show that BRG1 is recruited to DSBs to stimulate repair (Gong et al. 2015; Qi et al. 2015) and repress transcription for efficient repair to occur (Kakarougkas et al. 2014). We first examined the influence of BRG1 deficiency on ETO-induced cell apoptosis. SW13 cells, which are BRM and BRG1 deficient (Wong et al. 2000), were transfected with pBJ5-BRG1 or an empty vector. Then, the cells were treated with the indicated concentrations of ETO for 20 min, and cell apoptosis was analyzed by flow cytometry. Figure 1(A) shows that the percentage of apoptosis was lower in the SW13 cells expressing BRG1 than in the cells transfected with the empty vector. Next, the U2OS cells were transfected with BRG1-specific small interfering (si) RNA or control siRNA to knockdown BRG1 expression. These cells were treated with increasing concentrations of ETO, and cell apoptosis was detected. We found that U2OS cells transfected with BRG1 siRNA had a higher percentage of cell apoptosis than the control cells (Figure 1(B)). Taken together, these findings suggest that the absence of BRG1 leads to obvious ETO-induced cell apoptosis and renders cells sensitive to DNA damaging drugs.

### 3.2. BRG1 facilitates the DNA damage repair induced by ETO treatment

**Figure 2.**
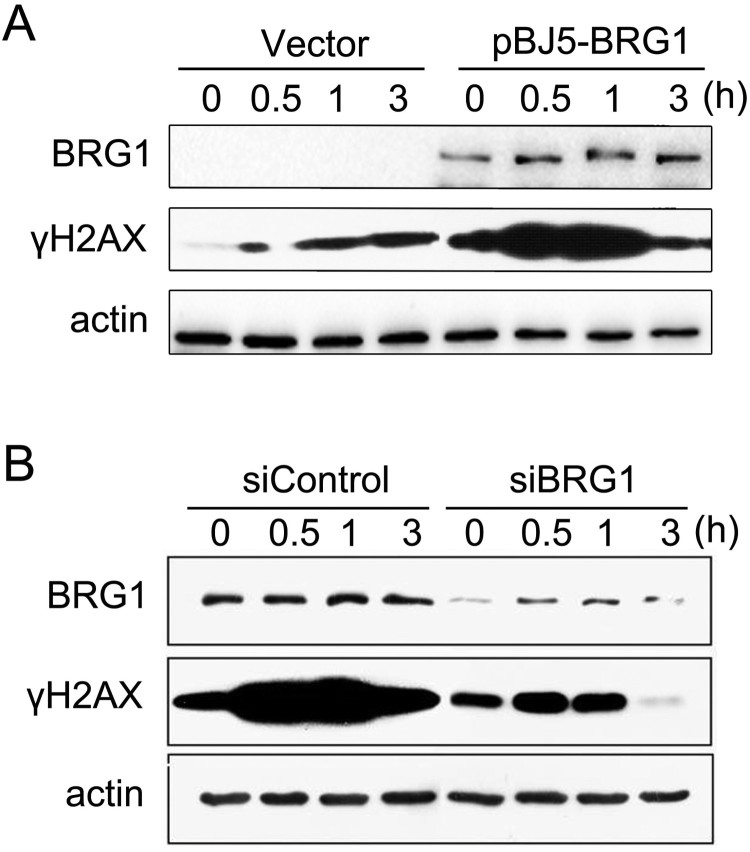
BRG1 promotes H2AX phosphorylation. (A) SW13 cells were transfected with the pBJ5 vector or pBJ5-BRG1 plasmids for 48 h. Then, the cells were treated with 10 μM ETO for 20 min and allowed to repair for the indicated time. The whole cell lysates were detected by western blotting with the indicated antibodies. (B) U2OS cells were transfected with control siRNA or BRG1 siRNA for 48 h. Then, the cells were treated with 10 μM ETO for 20 min and allowed to repair for the indicated time. The whole cell lysates were detected by western blotting with the indicated antibodies.

The histone variant H2AX, which is a sensor, is phosphorylated in response to DNA damage (Patne et al. 2017). We next explored whether the high cell apoptosis rate in BRG1-depleted cells after DNA damage was coupled with impaired DSB repair signaling. We examined the phosphorylation levels of H2AX following ETO treatment. SW13 cells transfected with pBJ5-BRG1 or the empty vector were treated with ETO for 20 min and allowed to repair. *γ*H2AX levels were detected by western blotting. Figure 2(A) shows that the level of *γ*H2AX was significantly increased (0.5 h and 1 h) in SW13 cells expressing BRG1, while the level of *γ*H2AX in the control cells was very low. We then depleted BRG1 expression in U2OS cells, treated them with ETO for 20 min and allowed them to repair. As shown in Figure 2(B), BRG1 knockdown cells had obviously lower H2AX phosphorylation levels than control cells. These data indicate that BRG1 is important for the phosphorylation of H2AX after DNA damage.

**Figure 3.**
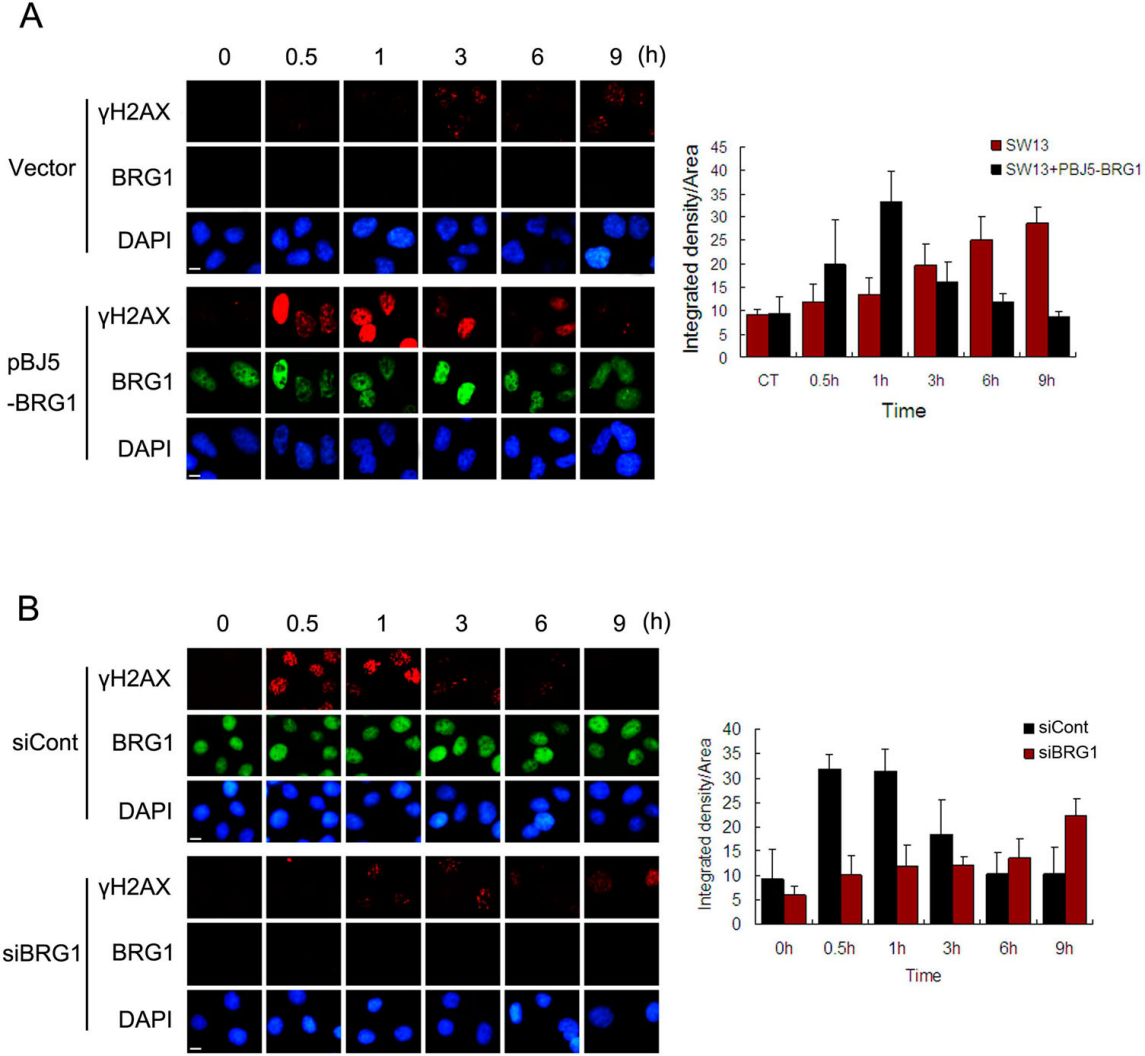
BRG1 knockdown impairs DNA damage repair. (A) SW13 cells were transfected with the pBJ5 vector or pBJ5-BRG1 plasmids for 48 h. Then, the cells were treated with 10 μM ETO for 20 min and allowed to repair for the indicated time. The cells were fixed, permeabilized and immunostained with BRG1 and *γ*H2AX antibodies. Images were captured with a fluorescence microscope. The relative *γ*H2AX levels were quantified by ImageJ software. Scale bar: 10 μm. (B) U2OS cells were transfected with control siRNA or BRG1 siRNA for 48 h. Then, the cells were treated with 10 μM ETO for 20 min and allowed to repair for the indicated time. The cells were fixed, permeabilized and immunostained with BRG1 and *γ*H2AX antibodies. Images were captured with a fluorescence microscope. The relative *γ*H2AX levels were quantified by ImageJ software. Scale bar: 10 μm. siCont: control siRNA. siBRG1: BRG1 siRNA.

Next, we detected the phosphorylation of H2AX in SW13 and U2OS cell after ETO treatment by immunofluorescence. SW13 cells expressing BRG1 showed obviously increased *γ*H2AX staining (1 h), which decreased to the background level at 9 h. Moreover, the phosphorylation of H2AX was delayed, and the dephosphorylation of *γ*H2AX was impaired in the control SW13 cells (Figure 3(A)). In addition, BRG1 knockdown reduced the level of *γ*H2AX staining at the early phase (0.5 h and 1 h) and delayed the clearance of *γ*H2AX staining at the later time points (3–9 h) in U2OS cells (Figure 3(B)). These findings suggest that BRG1 is essential for ETO-induced DNA damage response and repair.

### 3.3. BRG1 knockdown decreases the sensitivity of chromatin to MNase

**Figure 4.**
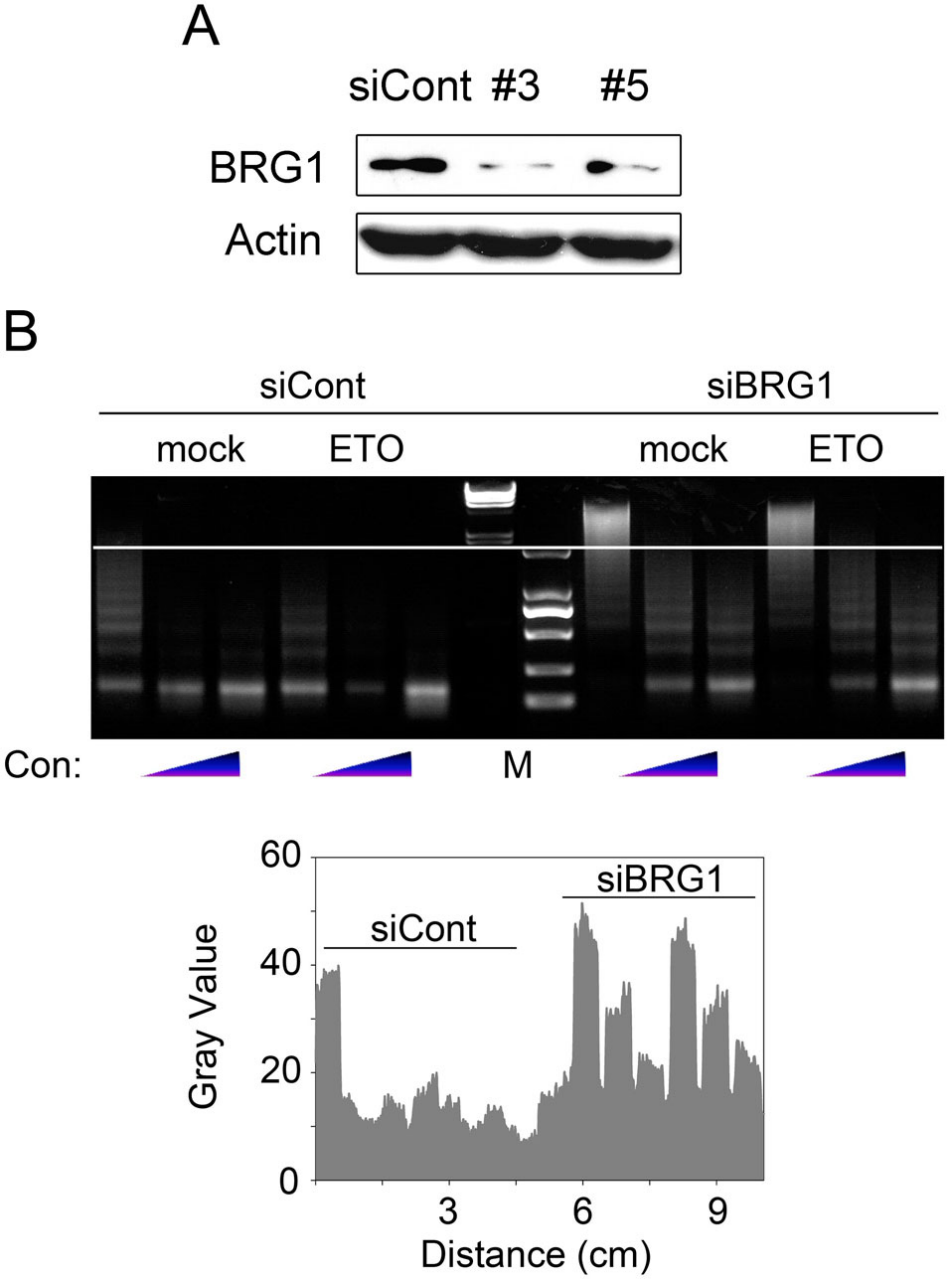
Loss of BRG1 protects chromatin from MNase digestion. (A) U2OS cells were transfected with control or two BRG1 siRNAs (#3 and #5). Forty-eight hours later, the cells were lysed and detected by western blotting with BRG1 and actin antibodies. (B) U2OS cells were transfected with siRNA #3 for 48 h. Then, the cells were treated with 10 μM ETO or not for 20 min. One hour later, the nuclei were digested with 2 U, 5 U or 10 U MNase for 5 min at room temperature. The MNase-treated nucleosomal DNA was resolved on 1% agarose gels and visualized with ethidium bromide (upper panel). The quantification of the DNA amount marked by the white line is shown in the lower panel. siCont: control siRNA. siBRG1: BRG1 siRNA. Con: concentration.

Given that the SWI/SNF family members can use ATP hydrolysis to evict H2A-H2B dimmers or even entire histone octamers (Osley et al. 2007), the enrichment of BRG1 at DSB sites is likely related to its function in chromatin structure modulation. Thus, we performed micrococcal nuclease susceptibility assays to test the influence of BRG1 on the global chromatin structure before and after DSB induction. Immunoblot analysis showed the efficacy of BRG1 depletion (Figure 4(A)). As shown in Figure 4(B), the cells transfected with control siRNA exhibited an increased lower molecular weight smear after ETO treatment, indicating an increased susceptibility to MNase after DNA damage. However, BRG1 knockdown resulted in a much higher molecular weight smear even after ETO treatment, suggesting that BRG1-depleted cells exhibited decreased genome digestion by MNase. These data indicate that BRG1 depletion led to a more condensed overall chromatin structure.

### 3.4. BRG1 promotes chromatin remodeling around DNA damage sites

**Figure 5.**
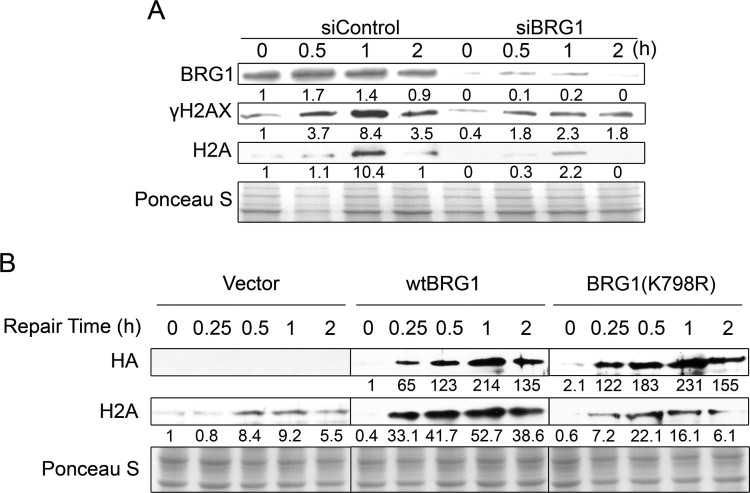
BRG1 depletion reduces chromatin relaxation near DSB sites. (A) U2OS cells expressing control siRNA or BRG1 siRNA #3 were incubated with 10 µM ETO. At the indicated repair times, the cell pellets were extracted in 1.0 M NaCl, and the released proteins were detected by western blotting analyses with the indicated antibodies. Ponceau S staining was used to demonstrate equal loading. (B) SW13 cells expressing HA-tagged wtBRG1, HA-tagged ATPase mutant BRG1 (K798R) or empty vector were treated with 10 μM ETO for 20 min and allowed to repair in fresh medium for 15, 30, 60 and 120 min. The cell pellets were extracted in 1.0 M NaCl, and the released histones were detected by western blotting analyses with the indicated antibodies. Ponceau S staining was used to demonstrate equal loading.

To further examine the role of BRG1 in nucleosome modulation around DNA damage, we performed nucleosome stability assays. U2OS cells transfected with BRG1 siRNA or control siRNA were treated with ETO and extracted with 0.15 M NaCl for nuclei isolation at the indicated repair time intervals. The isolated nuclei were further extracted in 1.0 M NaCl to release any chromatin-associated proteins. As shown in Figure 5(A), DNA damage increased the release of histone H2A from chromatin, and BRG1 knockdown significantly decreased the solubility of histone H2A in a high-salt solution. Moreover, in SW13 cells transfected with pBJ5-wtBRG1, ATPase mutant BRG1 (K798R) or empty vector, we found that the DNA damage-dependent increase in the solubility of histone H2A in high-salt extracts was enhanced greatly in SW13 cells expressing wtBRG1. However, we observed less H2A release in empty vector-transfected and mutant BRG1-transfected cells (Figure 5(B)).

**Figure 6.**
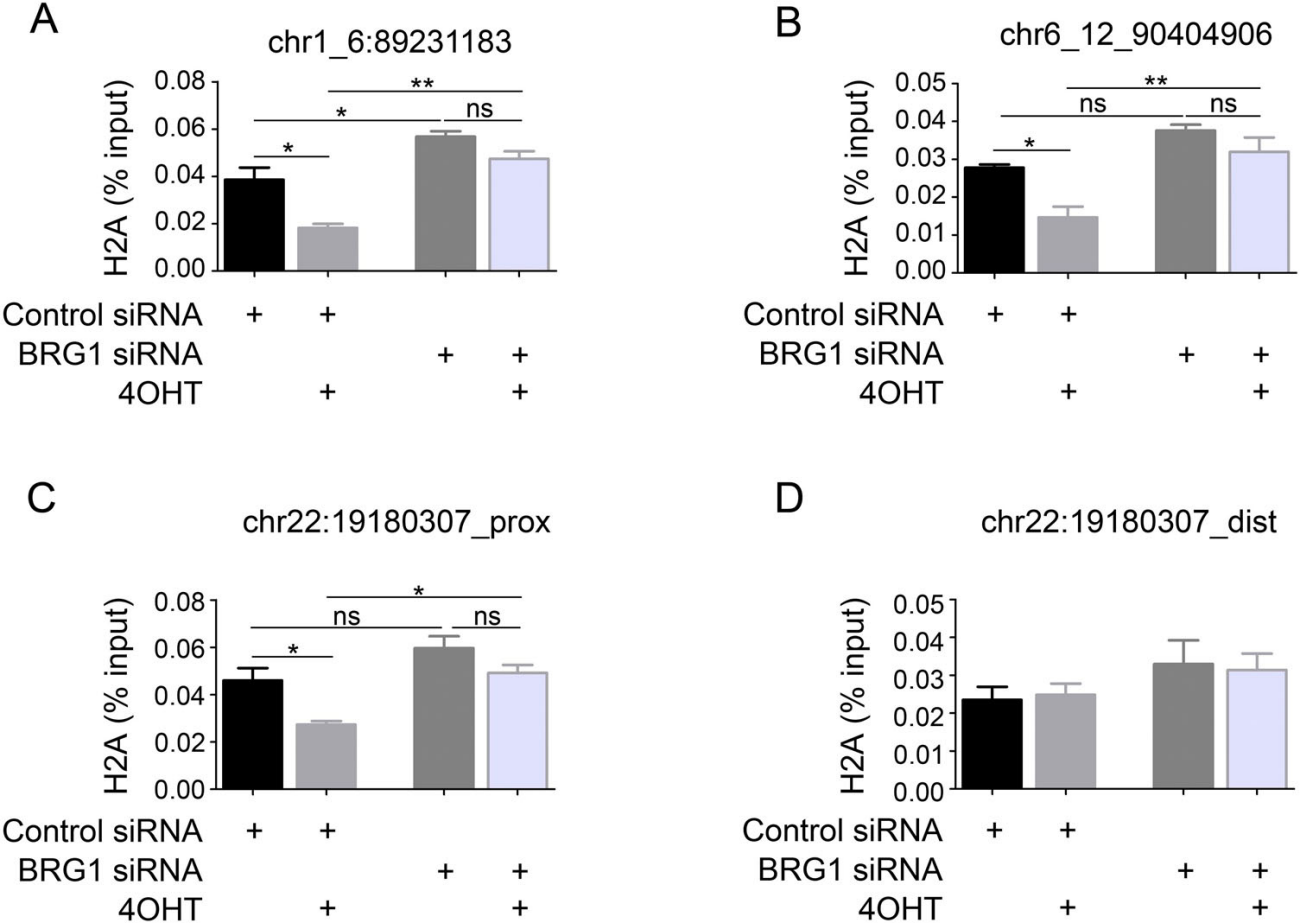
BRG1 promotes H2A release from chromatin near DSB sites. (A, B) H2A ChIP assays were performed on AsiSI-ER-U2OS cells treated with 4OHT or not for 4 h; the products were assessed by real-time q-PCR amplification using primers flanking the chr1_6:89231183 and chr6_12:90404906 DSB sites. Each value represents the mean ± S.D. of three independent experiments. (C, D) H2A ChIP assays were performed on AsiSI-ER-U2OS cells after treatment with 4OHT for 4 h or not; the products were assessed by real-time q-PCR amplification using primers near or distal to the chr22:19180307 DSB site. Each value represents the mean ± S.D. of three independent experiments.

To examine the release of H2A from the nucleosome at specific DSB sites, we utilized U2OS cells stably expressing the HA-*Asi*SI-ER enzyme, an 8-bp cutter that generates fragments longer than 1 mega bp on average in the human genome (Iacovoni et al. 2010). Fused *Asi*SI contains a modified estrogen receptor hormone-binding domain (ER), which binds to only 4OHT. Previous study has found that 4OHT treatment induces the nuclear localization of *Asi*SI-ER and generates DSBs at chr1_6:89231183, chr6_12:90404906 and chr22:19180307 *Asi*SI site (Littlewood et al. 1995; Agger et al. 2005; Iacovoni et al. 2010). We investigated the distribution of H2A around the specific 4OHT-induced DSBs by ChIP analysis using primers near the chr1_6:89231183 and chr6_12:90404906 DSB sites. As shown in Figure 6(A,B), BRG1 knockdown significantly decreased the dissociation of H2A from the chr1_6:89231183 and chr6_12:90404906 DSB sites. Furthermore, we detected the H2A distribution proximally (3.7 Kb) and distally (2 Mb) from the specific DSB site on chr22:19180307. We found that BRG1 knockdown significantly decreased the dissociation of H2A proximal, but not distal, to the DSBs (Figure 6(C,D)). The findings above indicate that BRG1 decreases the stability of the nucleosomes at DSBs and creates an open and relaxed chromatin structure.

## 4. Discussion

During DNA DSB repair, the condensed chromatin structure prevents repair factors from accessing the broken DNA. SWI/SNF has been defined as an important chromatin remodeler and transcriptional regulator in DSB repair (Brownlee et al. 2015). Recent studies using mammalian cells have shown that BRG1 can be recruited to DSBs by interacting with *γ*H2AX-containing nucleosomes (Lee et al. 2010). Previous work in our lab has shown that BRG1 participates primarily in homologous recombination repair by facilitating the replacement of RPA with RAD51 at DSB sites (Qi et al. 2015). In this study, we found that apoptosis was increased in BRG1-depleted cells after treatment with the DNA-damaging drug ETO. Moreover, BRG1 contributed to the relaxation of chromatin near DSB sites. Taken together, these results indicate that BRG1 plays multiple roles in the efficient execution of the DNA damage repair process.

Increasing data show that many chromatin remodelers are critical for altering the chromatin structure to promote DSB repair (Qi et al. 2016; Ooka et al. 2018; Zhou et al. 2018). In the present study, we observed histone H2A release from chromatin after empty vector-transfected and mutant BRG1-transfected cells were treated with ETO (Figure 3(B)). These data implied that there may be other factors involved in DSB-associated nucleosome modulation. The mechanisms underlying how these chromatin remodelers interact with each other in the DNA damage repair process need to be studied further.

Many data are available regarding gene mutations and DNA repair protein(s) expression in cancers (Ali et al. 2017), and BRG1 is mutated in many types of human cancer cell lines (St Pierre and Kadoch 2017). In light of these data, we believe that BRG1 could be a promising target for cancer prevention and therapy.
